# Impacts of residual 3D printing metal powders on immunological response and bone regeneration: an in vivo study

**DOI:** 10.1007/s10856-023-06727-1

**Published:** 2023-05-25

**Authors:** Jincheng Tang, Zhuo Sang, Xiaolei Zhang, Changhui Song, Wei Tang, Xiaoping Luo, Ming Yan

**Affiliations:** 1 Department of Materials Science and Engineering, Southern University of Science and Technology, Shenzhen, 518055 China; 2 The Eighth Affiliated Hospital, Sun Yat- sen University, Shenzhen, 518033 China; 3 Department of Mechanical and Automotive Engineering, South China University of Technology, Guangzhou, 510641 China; 4 Shenzhen Institutes of Advanced Technology, Chinese Academy of Sciences, Shenzhen, 518055 China; 5 Nanjing Stomatological Hospital Medical School of Nanjing University, Nanjing, 210008 China; 6 Jiaxing Research Institute, Southern University of Science and Technology, Jiaxing, 314001 China

**Keywords:** Powder bed fusion additive manufacturing, Metal powder, Immunological response, Osteolysis, Bone regeneration

## Abstract

Residual powder is a defect in powder bed fusion-based additive manufacturing (3D printing), and it is difficult to completely remove it from as-printed materials. In addition, it is not necessary to apply 3D printed implants with residual powder in the clinic. The immunological response triggered by the residual powder is an important area of study in medical research. To further understand the possible immunological reactions and hidden dangers caused by residual powders in vivo, this study compared the immunological reactions and osteolysis caused by typical powders for four implant materials: 316 L stainless steel, CoCrMo, CP-Ti, and Ti-6Al-4V (particle size range of 15–45 μm), in a mouse skull model. Furthermore, the possible immunological responses and bone regeneration induced by the four 3D printed implants with residual powder in a rat femur model were compared. In the mouse skull model, it was found that the 316L-S, CoCrMo-S, and especially the 316L-M powders, upregulated the expression of pro-inflammatory factors, increased the ratio of RANKL/OPG, and activated more functional osteoclasts, resulting in more severe bone resorption compared with those in other groups. In the rat femur model, which is more suitable for clinical practice, there is no bone resorption in implants with residual powders, but they show good bone regeneration and integration ability because of their original roughness. The results indicate that the expressions of inflammatory cytokines in all experimental groups were the same as those in the control group, showing good biological safety. The results answered some critical questions related to additively manufactured medical materials in vivo and indicated that as-printed implants may have great potential in future clinical applications.

**Graphical Abstract:**

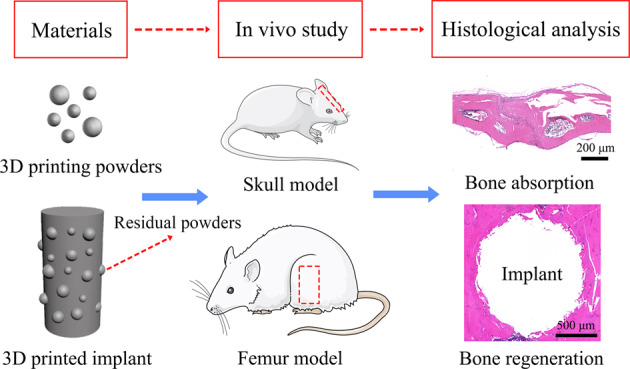

## Introduction

Medical materials that can replace damaged organs, tissues, blood vessels, or intact body parts are expected to be safe, reliable, and highly biocompatible in the long-term. Metallic medical materials were one of the earliest medical materials utilized by human beings, and their medical applications can be traced back to the industrial revolution era in the 19th century [1]. 316 L, CoCrMo, Ti, and Ti alloys are the most widely used load-bearing implants in clinical applications, and are mainly used for diagnosis, treatment, tissue replacement, and tissue enhancement [2–4]. Compared with other types of medical materials, metallic materials have excellent properties, such as high strength, good toughness, good bending fatigue resistance, excellent processability, and biocompatibility [5–7]. The rapid development of additive manufacturing (AM, also known as 3D printing) technology has led to the wide use of metallic medical materials such as vascular stents, catheter guide wires, fractured internal fixation plates, screws, artificial joints, and dental implants [8].

3D metal printing technology, including electron beam melting (EBM), laser-engineered net shaping (LENS), and selective laser melting (SLM), is an important development direction for advanced manufacturing technology. In particular, SLM technology has attracted significant interest because of its unique capabilities to achieve near-net shape and customization of metal implants; however, it also has some defects, such as excessive holes, warpage, residual stress, and residual powder [9–11]. Residual powders, either as loose powders or partially melted powders, are commonly found in as-printed materials, as shown in Fig. S1 [12–14]. In the SLM process, insufficient energy input and/or powder spatter are the main reasons for partial melting and loosening of the powder [15, 16]. Common post-treatment methods, such as chemical polishing, pickling, and high-pressure jetting, can remove most of the residual powder on the surface but may not remove the residual powder inside the as-printed porous implant [17–19]. Therefore, the immunological response triggered by the residual powder is an important area of study in medical research.

In previous studies, the immunological behavior induced by implants was mainly aimed at the ions and wear particles (caused by biological corrosion and mechanical motion friction) released by the implants. During long-term implantation, the released ions are transported away from the implantation site through the body’s circulatory system, causing bone resorption and aggravating inflammatory responses in bone and other tissues [20, 21]. In addition, the wear particles produced by metal implants during service can activate monocytes and macrophages in granuloma tissue, enhance osteoclast development and activity, inhibit osteoblast activity, and induce bone resorption [22, 23]. In our previous study, it was found that metallic 3D printing powders have more difficulty triggering immunological responses than ions and wear particles because of their regular shape, size, and surface chemical composition [24].

In summary, for the clinical application of as-printed implants, a more systematic understanding of the immunological response to metal powders is necessary. In this study, we investigated the expression of related cytokines in vitro and induced bone resorption of metal powders of various particle sizes in vivo. In particular, the immunological response and bone regeneration induced by implants with residual powder particles in vivo were further investigated. As a result, this study contributes to a better understanding of how residual metal powder particles may affect patients.

## Materials and methods

### Preparation and characterization of powder particles

**Fig. 1:**
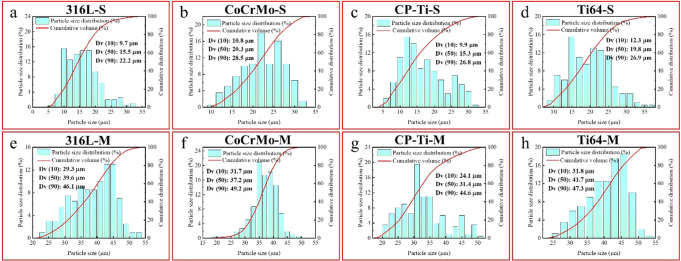
Particle sizes distribution and cumulative volume of different powders for (**a**) 316L-S,(**b**) CoCrMo-S, (**c**) CP-Ti-S, (**d**) Ti64-S, (**e**) 316L-M, (**f**) CoCrMo-M, (**g**) CP-Ti-M,and (**h**) Ti64-M, respectively

316 L stainless steel, CoCrMo alloy, commercially-pure Ti, and Ti-6Al-4V atomized metallic powders were sieved into two size groups, ≤32 μm and 32–45 μm, using a Retesch shaker. Because many AM processes (such as EBM and SLM) employ metal powder in the 15–100 μm range, these sizes were selected. In particular, our previous study has found that powder particles in the range of 75–100 μm did not cause changes in the expression of inflammatory factors related to immune cells in vitro [24]. The samples will be referred to as 316L-S, 316L-M, CoCrMo-S, CoCrMo-M, CP-Ti-S, CP-Ti-M, Ti64-S, and Ti64-M, respectively. Their particle size distribution and cumulative volume are summarized in Fig. 1.

A scanning electron microscopy (SEM, SU820, Japan) with a 5 kV accelerating voltage was used to characterize the powders. The powders were analyzed for particle size using Image J software. The diameters of the volume-equivalent spheres were calculated using the following formula below to derive the volume-based particle sizes [25]:

1 $$D_V = \left( {\frac{6}{\pi }V_P} \right)^{\frac{1}{3}}$$

where *D*_*v*_ is the diameter of a volume-equivalent sphere and *V*_*p*_ is the volume of the particle.

For endotoxin removal, all sieved powder particles were repeatedly washed with a mixture of 0.1 N sodium hydroxide and 25% nitric acid, followed by autoclaving for 20 min at 120 °C. After sterilization, the particles were washed three times with sterile phosphate buffer solution (PBS), and re-suspended with sterile saline to a final concentration of 1 × 10^6^ particles/mL. The suspension is placed in a refrigerator at 4 °C for use. In this study, the weight of powder particles at this concentration was calculated using the following formula:

2 $$m_p = \frac{{N_p\,\rho _p\,\pi D_{v50}^3}}{6}$$

where *m*_*p*_ is the weight of powder particles, *N*_*p*_ is the number of particles, *ρ*_*p*_ is the effective density of powder particles, and *D*_*v50*_ is average diameter of a volume-equivalent sphere.

### Preparation and characterization of 3D printed implants with residual powders

Gas atomized powders with a size range of 15–53 μm were used in this study. An SLM® 125HL 3D printer with a 400 W IPG fiber laser (SLM Solutions Group AG, Germany) was employed to fabricate in vivo implants (Φ 1.5 × 2 mm). The laser wavelength and spot diameter were 1070 nm and 65 μm, respectively. 316 L, CoCrMo, CP-Ti, and Ti64 implants were printed and shown in Table S1. During printing, a high purity argon atmosphere is provided in the printing chamber. The 3D printed CP-Ti implants polished with 2000 grift were designated as the control group (referred to as CP-Ti-P), and other implants were simply ultrasonically cleaned for 20 min. The surface morphologies of the implants were characterized using SEM with a 5 kV accelerating voltage.

### Cell culture

Raw 264.7 cells (ATCC, USA), a murine macrophage cell line, were cultured in 55 cm^2^ tissue culture polystyrene (TCP) with High-DMEM medium containing 10% fetal bovine serum (FBS, Gibco), 1% penicillin and streptomycin. The cells were incubated in a CO_2_ incubator with 5% CO_2_ and 95% humidity at 37 °C, and the culture medium was refreshed every three days.

### Enzyme-linked immunosorbent assay (ELISA) analysis

The above metal powders (approximately 1 × 10^6^ particles/mL concentration) were cultured with Raw 264.7 cells planted at a density of 2 × 10^4^ per well in 48-well TCPs for 7 days. After collecting the medium, it was centrifuged for 10 min at 2500 rpm. The medium’s supernatant was retrieved and stored at −80 °C. ELISA kits (Jiangsu Jingmei Biological Technology Co., Ltd, China) were used to measure the expression levels of pro-inflammatory (TNF-α and IFN-γ) and anti-inflammatory (IL-10 and TGF-1) cytokines in macrophages cultured with various powder particles. It should be noted that the four types of cytokines studied here are widely studied in both clinical cases and scientific researches [26]. In the background of bone issue response [27], TNF-α and IFN-γ, the two pro-inflammatory factors, mainly mediate cellular immunity. Their increased expression induces macrophage activation, which in turn accelerates bone tissue resorption. TGF-β and IL-10 are also involved in the bone tissue response. They tend to promote fibrosis and osteogenic differentiation, thereby shortening bone repair time. They are important expressions to understand the immune behavior induced by the metal powders. Finally, an enzyme indicator was used to detect its absorbance at 450 nm.

### Mouse skull model

#### Animals and grouping

All animal operations and studies were authorized by the Animal Ethics Committee of the Southern University of Science and Technology, and laboratory animal care was performed in accordance with national guidelines (Approval No. SUSTC-2020-121). Specific pathogen-free (SPF)-grade healthy C57BL/6 male mice aged 10 weeks (average weight: 20–22 g) were randomly assigned into nine groups (*n* = 6 per group): control (injected with sterile saline), 316L-S, 316L-M, CoCrMo-S, CoCrMo-M, CP-Ti-S, CP-Ti-M, Ti64-S, and Ti64-M groups. All experimental groups were injected with sterile saline containing powdered particles.

#### Surgical procedure

A skull model was established and carried out under sterile conditions. In brief, the mice were anesthetized by inhalation of isoflurane (maintenance concentration of 2%). After anesthesia, Meloxicam (1 μL g^−1^ body weight) was administered subcutaneously to provide surgical analgesia. The head was shaved and sterilized, and a median incision of 10 × 5 mm was made by incising along the median sagittal suture of the skull with a scalpel, and the periosteum of the skull was preserved. Then, 20 μL of suspended salt solution containing metal powder particles (1 × 10^6^ particles/mL) was taken during mixed shaking. It was then injected into the subperiosteum of the skull in the middle of the incision, and the incision was sutured layer by layer. On the second day after the operation, sterile saline at a dose of 5 mL/Kg was continuously administered for 14 days to maintain the osmotic pressure balance of body fluids. After 14 days, the mice were euthanized by cervical dislocation, and the skull was harvested. Three harvested skulls were randomly selected from each group and placed in a 1.5 mL centrifuge tube for subsequent ELISA analysis. In particular, other skulls from each group were used for further micro-CT and histological analysis.

#### ELISA analysis of cultured skull tissues

The obtained skull tissues were placed in a 6-well TCP plate with 2 mL of High-DMEM containing 1% penicillin and streptomycin, and incubated in a 5% CO_2_ incubator for 24 h. After incubation, the medium was centrifuged (1500 rpm, 15 min) and the supernatant was collected. The supernatant was diluted 5 fold and measured using ELISA kits following the manufacturer’s protocol.

#### Micro-CT analysis

The obtained skull tissues were fixed with 4% paraformaldehyde for 6 h and scanned using Micro-CT (Skyscan 1172, Bruker, Germany) under a source voltage of 100 kV, current of 200 μA, and an exposure time of 650 ms. NRecon software was used to perform three-dimensional (3D) reconstructions when the scan was completed. A 3 × 3 × 1 mm region of interest (ROI) was selected centering on the intersection of sagittal suture and coronal suture, and the bone indices of the area were further analyzed using the associated program CTAn software, including bone mineral density (BMD), bone volume fraction (BV/TV), number of pores, and area of pores.

#### Histological and immunohistochemical analysis

The skulls were preserved with 4% paraformaldehyde and hard tissue staining was performed (*n* = 3 per group). A complete decalcification of all samples was performed in 10% (w/v) EDTA (pH = 7.4) over a period of 3 weeks, followed by dehydration and embedding in paraffin. The coronal plane was used to segment the skull tissues, which were subsequently cut into five-micron thick sections. The tissue sections were stained with haematoxylin and eosin (H&E), TRAP, and immunohistochemical staining, and the sections were viewed and imaged using an automatic digital slide scanner (Pannoramic MIDI, 3D HISTECH, Hungary). Images of the ROI of the skull were acquired at low- and high -magnifications. The count method was used to determine the number of TRAP-positive multinucleated osteoclasts in the ROI of five consecutive sections. Image J was used to analyze the osteoclast surface per bone surface (OcS/BS, %) in each sample, as described previously [28, 29]. For immunochemical staining of RANKL and OPG, the positive cells were counted in the ROI of five consecutive sections and quantified using the Image J, as described previously [30].

### Rat femur model

#### Animals and grouping

All animal operations and studies under the model were also authorized by the Animal Ethics Committee of the Southern University of Science and Technology (Approval No. SUSTech-SL2021041401). SPF-grade healthy sprague dawley male rats aged 8 weeks (average weight: 350–400 g) were randomly assigned to five groups (*n* = 3 per group): CP-Ti-P (control group), 316 L, CoCrMo, CP-Ti, and Ti64.

#### Surgical procedure

The surgery was carried out under sterile conditions. In brief, the rats were anesthetized by inhalation of isoflurane (maintenance concentration of 3%). After anesthesia, Meloxicam (1 μL g^−1^ body weight) was administered subcutaneously to provide surgical analgesia. The right hind limb was shaved and sterilized, and a 10 × 5 mm incision was made by incising the skin and muscle above the femur to expose the distal femoral metaphysis. Drilling using a 1.5 mm drill bit created a cylindrical bone defect 1.5 mm in diameter and 2 mm in depth, and the shattered bone fragments were washed away with sterile saline. After inserting the sterilized implant into the bone deficiency, the incision was sutured layer by layer. Intramuscular injections of penicillin and oral administration of carprofen were administered at a dose of 6000 U kg^−1^ and 4 mg kg^−1^ during surgery and postoperatively for 3 days. After 8 weeks, the abdominal aortic blood of the animals was collected after anesthesia and used for ELISA detection, as described previously [31]. Finally, the animals were euthanized and the implants with surrounding tissues were harvested for further micro-CT and histological analysis.

#### ELISA analysis of blood

The collected blood was centrifuged (3000 rpm, 20 min) at 4 °C, and the separated serum was stored at −80 °C. The expression levels of inflammatory cytokines and CTX-1 protein were measured using ELISA kits following the manufacturer’s protocol.

#### Micro-CT analysis

For each euthanized rat, the harvested right femur was fixed in 4% paraformaldehyde. Then, the distal femur was scanned using High-precision Diondo-d2 (Diondo GmbH, Germany) under a source voltage of 120 kV, current of 100 μA, and an exposure time of 2000 ms. It should be noted that the CT equipment employed here has a better spatial resolution than the one used for the aforementioned mouse skull model study. The corresponding reason is due to the necessity to detail the bone tissue response to the implants, see results in the following Fig. 11. A 139 μm pixel flat panel detector with a 3072 × 3072 pixels resolution was used to collect projections of the femur as it was rotated over 360 degrees in equal increments. After the scan was completed, the implant and its peripheral bone tissue of 300 μm were reconstructed by using VOLUME GRAPHICS 3.3 software. The osteogenesis indices, including BMD, BV/TV, trabecular separation (Tb.Sp), trabecular number (Tb.N), and trabecular thickness (Tb.Th), were quantitatively analyzed for the peripheral bone tissue.

#### Histological and immunohistochemical analysis

The rat femurs were fixed with 4% paraformaldehyde and hard tissue staining was carried out (*n* = 3 per group). The implants in the remaining samples were removed after 3 weeks of complete decalcification in 10% (w/v) EDTA (pH = 7.4), and the samples were dehydrated, embedded in paraffin, and cut into five-micron thick sections. Each femur was sliced longitudinally into three sections (vertical to the implant in the femur condyle). All staining (including Masson staining) images were obtained and quantitative data were analyzed in the same way as in the previous chapter, with the peripheral 150 μm around the implants was defined as the ROI.

### Statistical analysis

All data were analyzed using one-way ANOVA of the SPSS 17.0 software (SPSS, Chicago IL, USA) and presented as mean ± standard deviation. Statistical values were indicated in the relevant experiments performed triplet at least. The statistical significance was defined as *p* < 0.05.

### Flowchart of the overall study

**Fig. 2:**
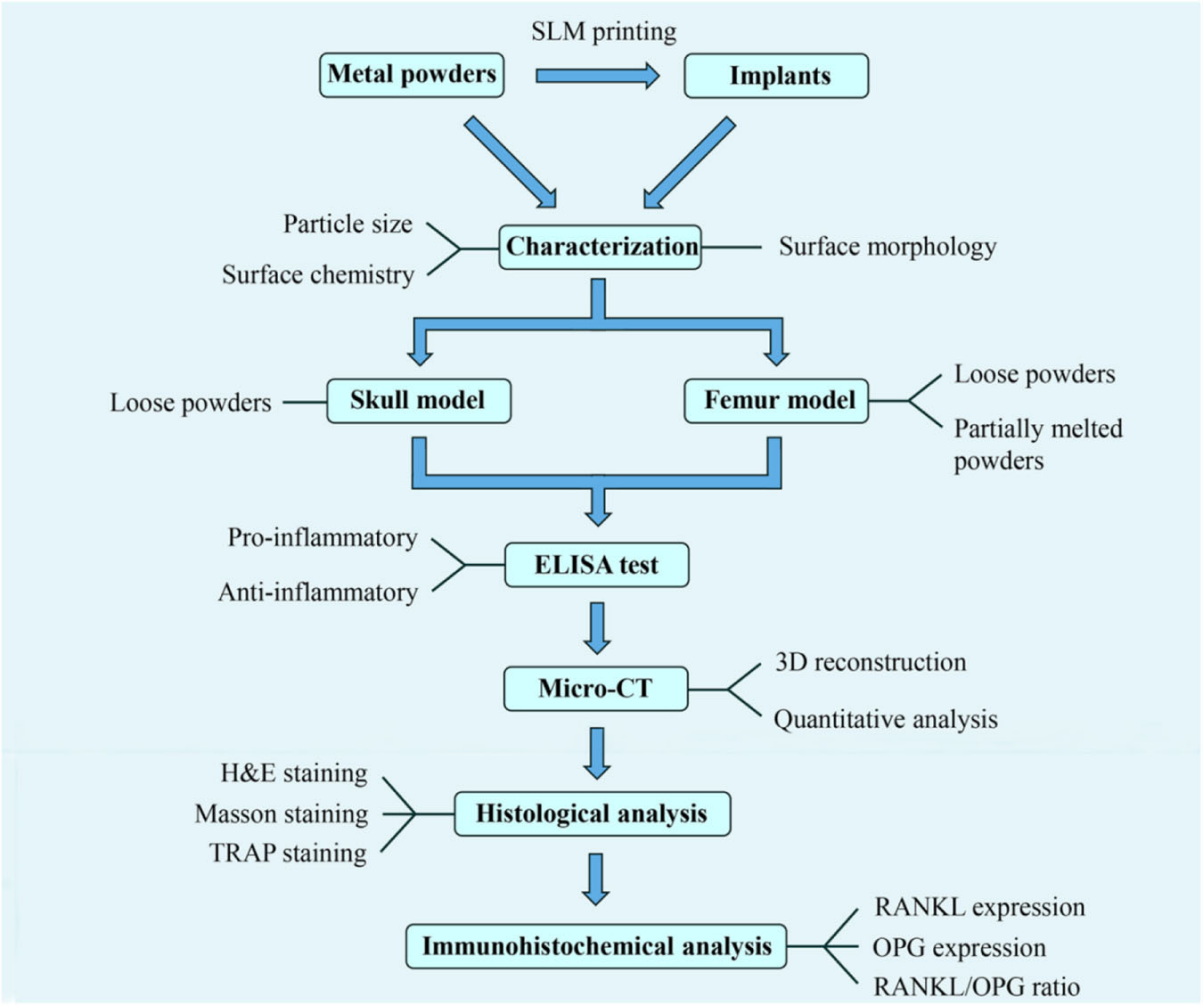
Flowchart of the entire study

The overall idea and flowchart of this study are shown in Fig. 2 for better understanding.

## Results

### Surface morphology of the metal powders and 3D printed implants

**Fig. 3:**
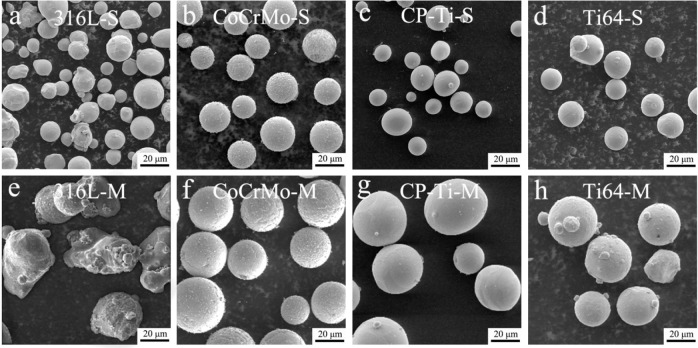
SEM micrographs of all powders with (**a**–**d**) small and (**e**–**f**) medium particle sizes

Figure 3 shows SEM micrographs of the 316 L, CoCrMo, CP-Ti, and Ti64 powders with various particle sizes. Based on the micrographs, it appears that the CoCrMo, CP-Ti, and Ti64 powders are spherical, whereas the 316 L powders are relatively non-spherical. Satellite powders are often attached to the surface of powders with medium particle sizes, particularly the 316L-M and CoCrMo-M groups, which is considered to be a typical feature of powders produced by the atomization process [32, 33].

**Fig. 4:**
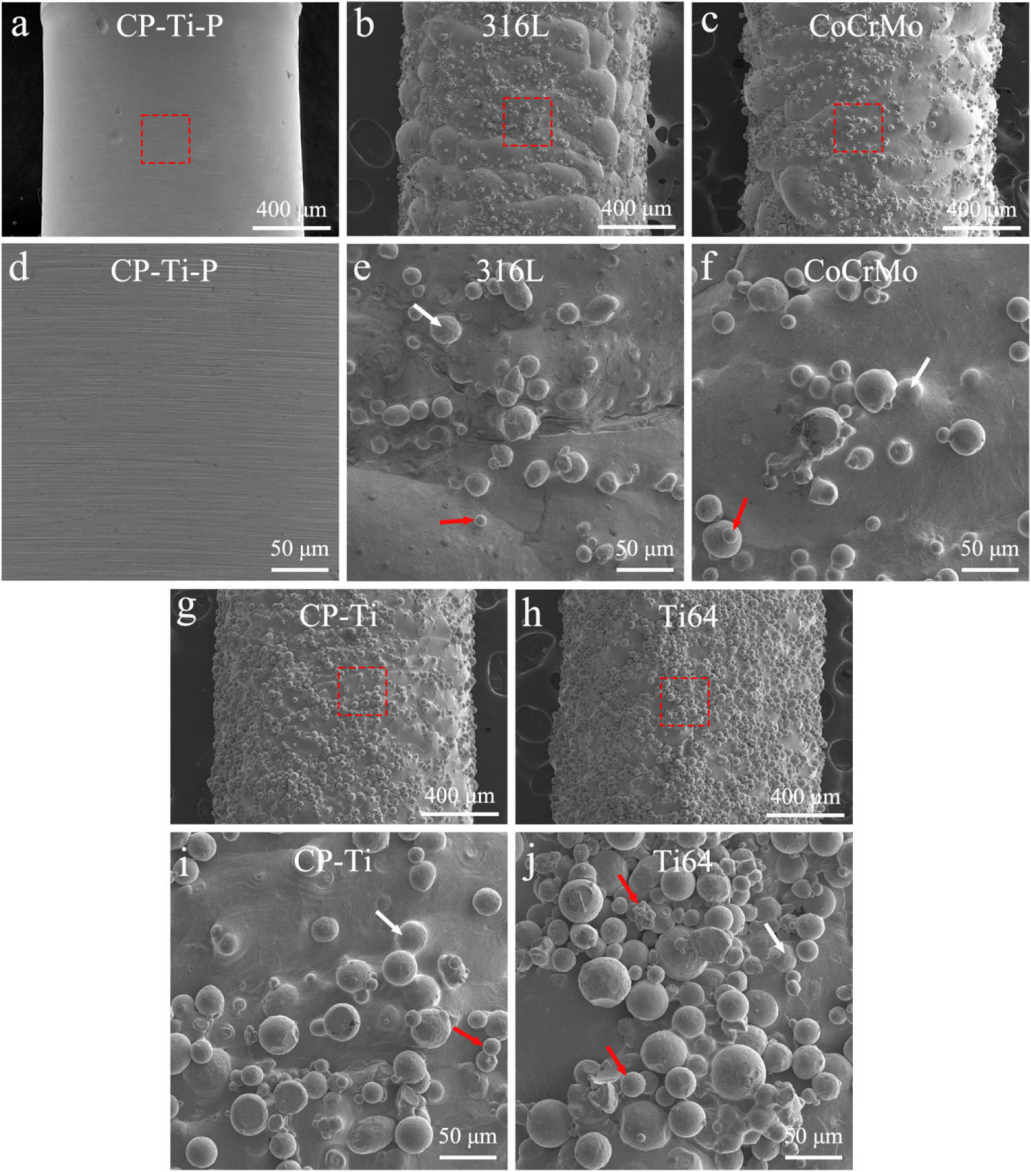
SEM morphology images of the 3D printed (**a**) CP-Ti-P, (**b**) 316 L, (**c**) CoCrMo, (**g**) CP-Ti, and (**h**) Ti64 implants. (**d**–**f**) Enlarged image of the red square area from (**a**), (**b**), and (**c**). (**i**, **j**) Enlarged image of the red square area form (**g**) and (**h**). Partially-melted powders (white arrows) and loose powders (red arrows) on the surface of the implants can be observed

Figure 4 presents the surface morphology of 3D printed CP-Ti-P, 316 L, CoCrMo, CP-Ti, and Ti64 implants. The surface of the CP-Ti-P implant after grinding was relatively smooth with a round contour. Whereas the surfaces of the other implants treated only by sonication were rough, the contour was nearly round, and a large amount of loose powders and partially melted powders were observed, especially for the Ti64 implant (Fig. 4h and j).

### Secretion of pro-inflammatory and anti-inflammatory cytokines in vitro and in vivo

**Fig. 5:**
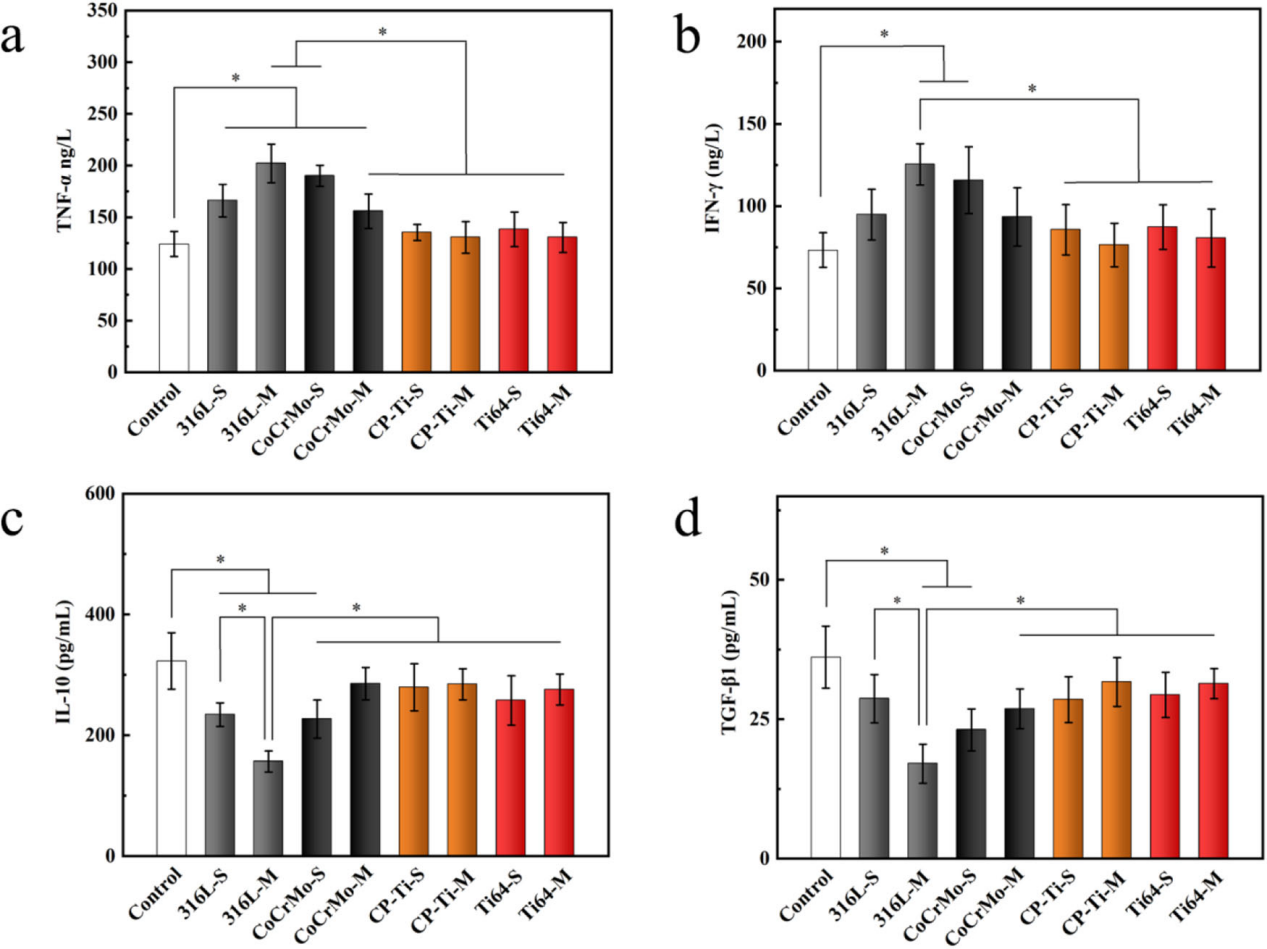
Expression of inflammatory cytokines in Raw 264.7 cells co-cultured with various powders for 7 days. **a** TNF-α, (**b**) IFN-γ, (**c**) IL-10, and (**d**) TGF-β1 (*p < 0.05)

The long-term immunological response was evaluated by the expression of cytokines produced by cells co-cultured with various metal powders of various particle sizes for 7 days. As expected, the expression of the pro-inflammatory cytokines TNF-α and IFN-γ and the 316L-M and CoCrMo-S groups was significantly upregulated by 2-fold compared with the control group value (Fig. 5a and b). The CP-Ti and Ti64 groups maintained the same levels as those of the control group. As for the anti-inflammatory cytokine IL-10, the expression in the 316L-M group was the lowest and was significantly higher than that in the other groups (Fig. 5c). Regarding the anti-inflammatory cytokine TGF-β1, the 316L-M and CoCrMo-S groups were significantly downregulated by 2-fold compared with that in the control group (Fig. 5d). The expression of all anti-inflammatory factors in the CP-Ti and Ti64 groups was similar to that in the control group.

**Fig. 6:**
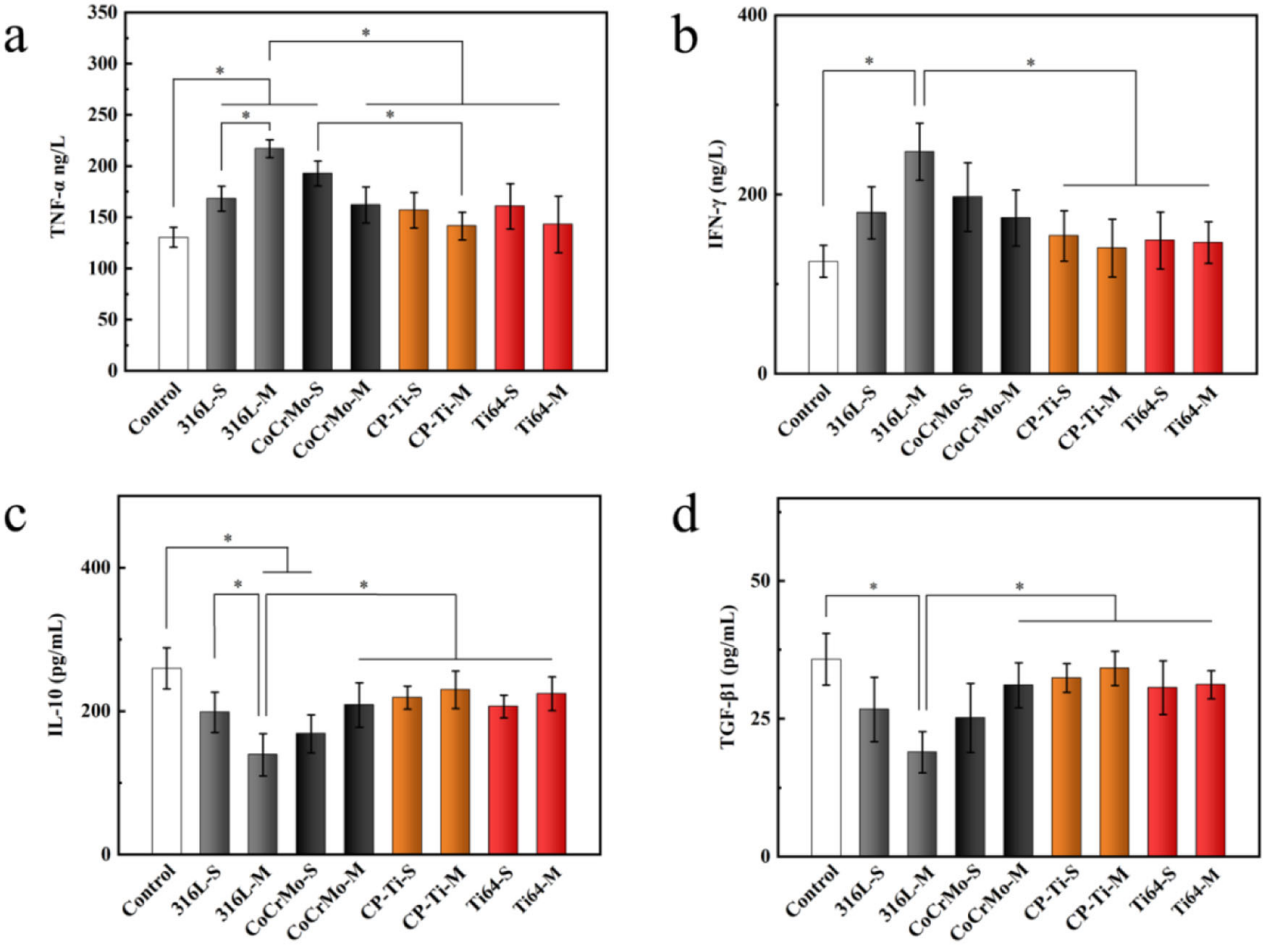
Expression of inflammatory cytokines in the supernatants of cultured skulls implanted with various powders for 24 h. **a** TNF-α, (**b**) IFN-γ, (**c**) IL-10, and (**d**) TGF-β1 (*p < 0.05)

To further evaluate the immunological response triggered by all powders in vivo, the levels of the inflammatory cytokines TNF-α, IFN-γ, IL-10, and TGF-β1 in the skull culture medium were detected by ELISA. Among the pro-inflammatory cytokines, the 316L-M group had the highest expression level, which was significantly higher than that of the other groups, except for that in the CoCrMo-S group (Fig. 6a and b). Similarly, the expression of anti-inflammatory cytokines in the 316L-M group was the lowest, and there was a significant difference compared to the other groups, except for the CoCrMo-S group (Fig. 6c and d). This indicates that exposure to 316L-M powder triggers immunological responses. The results indicate that the expressions of inflammatory cytokines in the Ti powder groups were the same as those in the control group, showing better biological safety. It is worth noting that there was no significant difference in the expression of inflammatory cytokines between the CoCrMo-S and CoCrMo-M groups; however, the expression levels of pro-inflammatory cytokines in the CoCrMo-S group were higher than those in the control group, and the expression of anti-inflammatory factors was lower than that in the control group. This indicated that the CoCrMo-S powder triggered an inflammatory response. In summary, the results were consistent with the results of the in vitro ELISA assay.

### Osteolysis and osteoclastogenesis triggered by metal powder particles

**Fig. 7:**
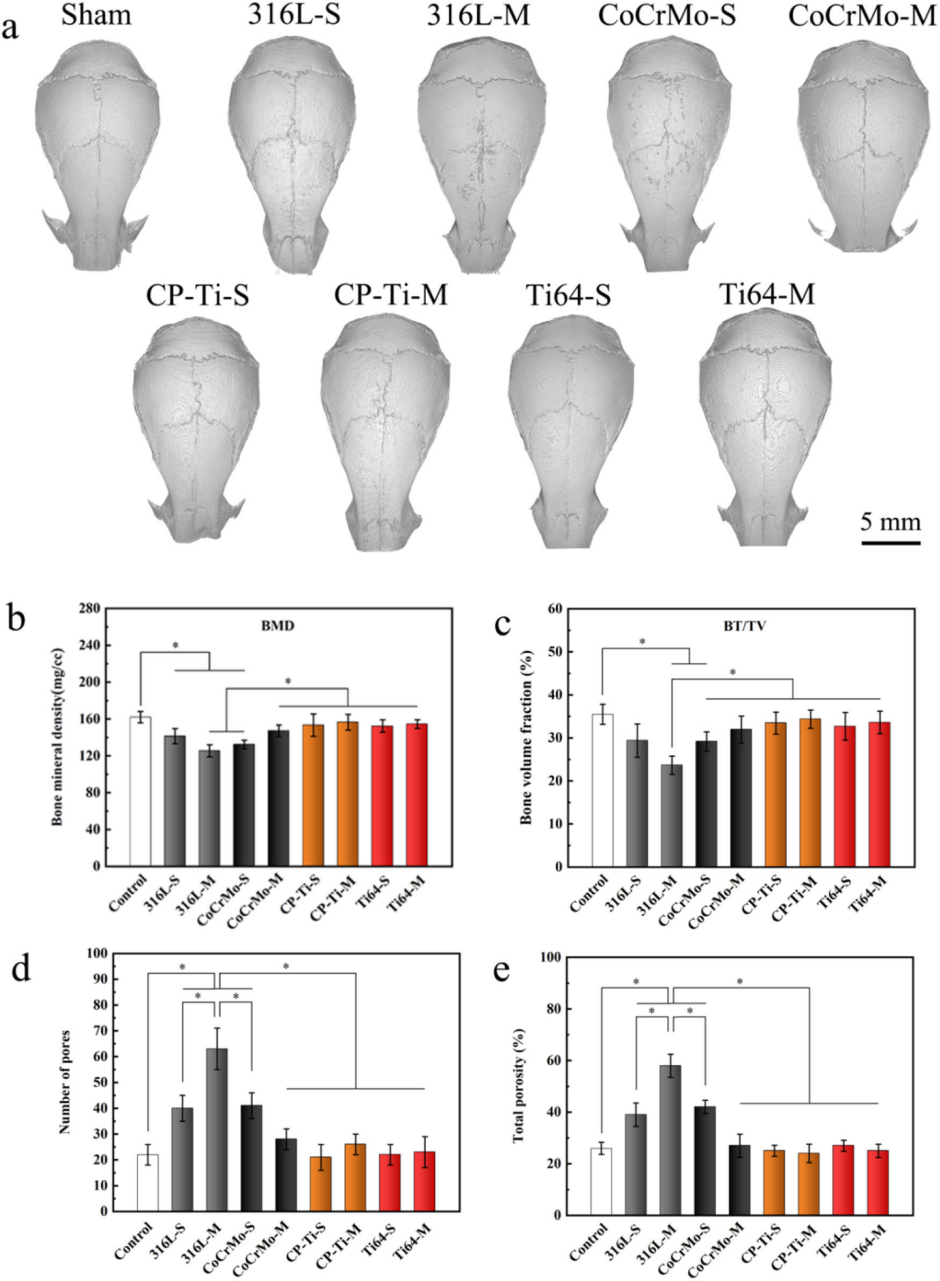
Osteolysis of mouse skull triggered by metal powders with various particle sizes. **a** 3D reconstructed images of the skull in each group. Quantitative analyses of the ROI, including (**b**) BMD, (**c**) BV/TV, (**d**) number of pores, and (**e**) total porosity (*p < 0.05)

The established mouse skull model was used to explore the osteolysis caused by the 3D printed metal powders in vivo. The 3D reconstruction results of micro-CT showed bone destruction, a rough bone surface, and a large number of skull lacunae in the implanted area of the 316L-M powder in mice (Fig. 7a). A small number of lacunae were also found on the skull surfaces of the 316L-S and CoCrMo-S groups. In contrast, the skulls of the control, CoCrMo-M, CP-Ti-S, CP-Ti-M, Ti64-S, and Ti64-M groups were smooth and intact, with no sign of osteolysis. Quantitative analysis showed that the BMD and BV/TV of the skull in the 316L-M and CoCrMo-S groups were significantly lower than those in the control group, whereas there was no significant difference between the Ti powder groups and the control group (Fig. 7b and c). The number of pores and total porosity of the 316L-S, 316L-M, and CoCrMo-S groups were significantly higher than those of the other groups, which is consistent with the reconstructed image features (Fig. 7d and e).

**Fig. 8:**
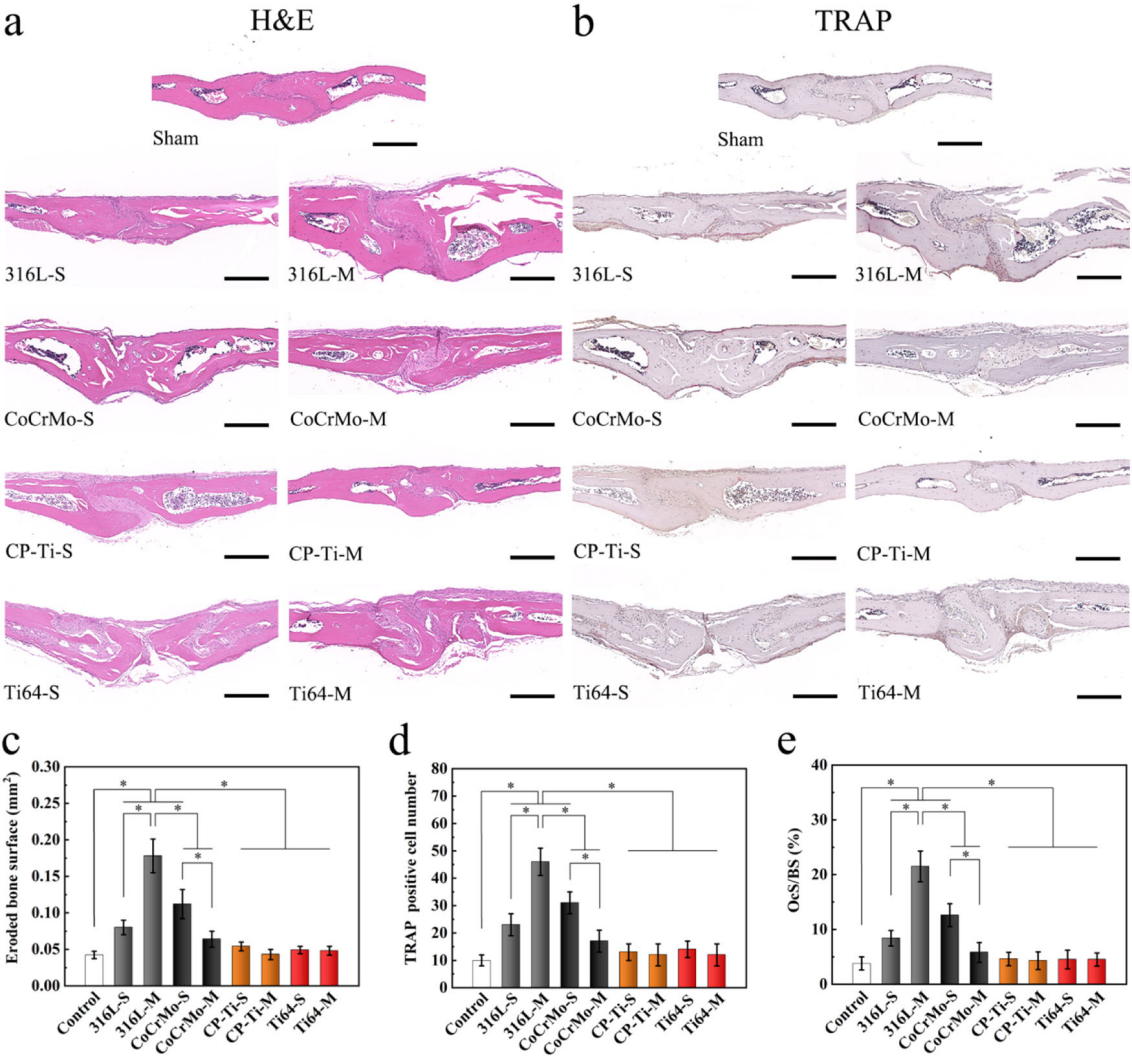
Histological analysis of skull sections. **a** H&E and (**b**) TRAP staining images of the ROI in each group (Scar bar = 200 μm). **c** Eroded bone surface (EBS, mm^2^), (**d**) the number of TRAP-positive multinucleated osteoclasts (dark red), and (**e**) percentage of the osteoclast surface per bone surface (OcS/BS, %) within the ROI in each group were measured (*p < 0.05)

To further analyze the increase in the number of osteoclasts induced by metal powders from 3D printing, which promote inflammation and ultimately lead to osteolysis, H&E and TRAP staining were used to analyze the pathological changes in the skull. The staining results showed obvious erosion on the top of the skull of the 316L-S, 316L-M, and CoCrMo-S groups in the implantation area of the metal powder, especially in the 316L-M group. Furthermore, a large number of inflammatory cells were observed in the bone destruction area, including lymphocytes, macrophages, and some osteoclasts (Fig. 8a and b). Based on the bone histomorphometric analysis, the EBS of the 316L-M group was the largest and was significantly higher than that of the other groups (Fig. 8c). In addition, the EBS of the 316L-S and CoCrMo-S groups was higher than that of the control group, but not that of the Ti powder groups.

TRAP-stained sections showed a large accumulation of TRAP-positive osteoclasts near the bone erosion surface in the 316L-S, 316L-M, and CoCrMo-S groups, especially in the 316L-M group. Quantitative analysis showed that the number of osteoclasts and OcS/BS of the 316L-M group were the highest, at 40 and 20%, respectively, significantly higher than those in the other groups (Fig. 8d and e). These results suggest that the 316L-S, 316L-M, and CoCrMo-S powders, especially the 316L-M powder, can promote the formation of osteoclasts, triggering serious inflammation and bone resorption, while the Ti powder has minimal effects.

### The RANK/OPG balance in vivo skull model

Recent studies have shown that RANKL/OPG balance plays a key role in osteoblast or osteoclast activation mediated by bone marrow stromal cells [34]. Therefore, immunohistochemical staining and skull analysis were performed to evaluate the expression of RANKL and OPG.

**Fig. 9:**
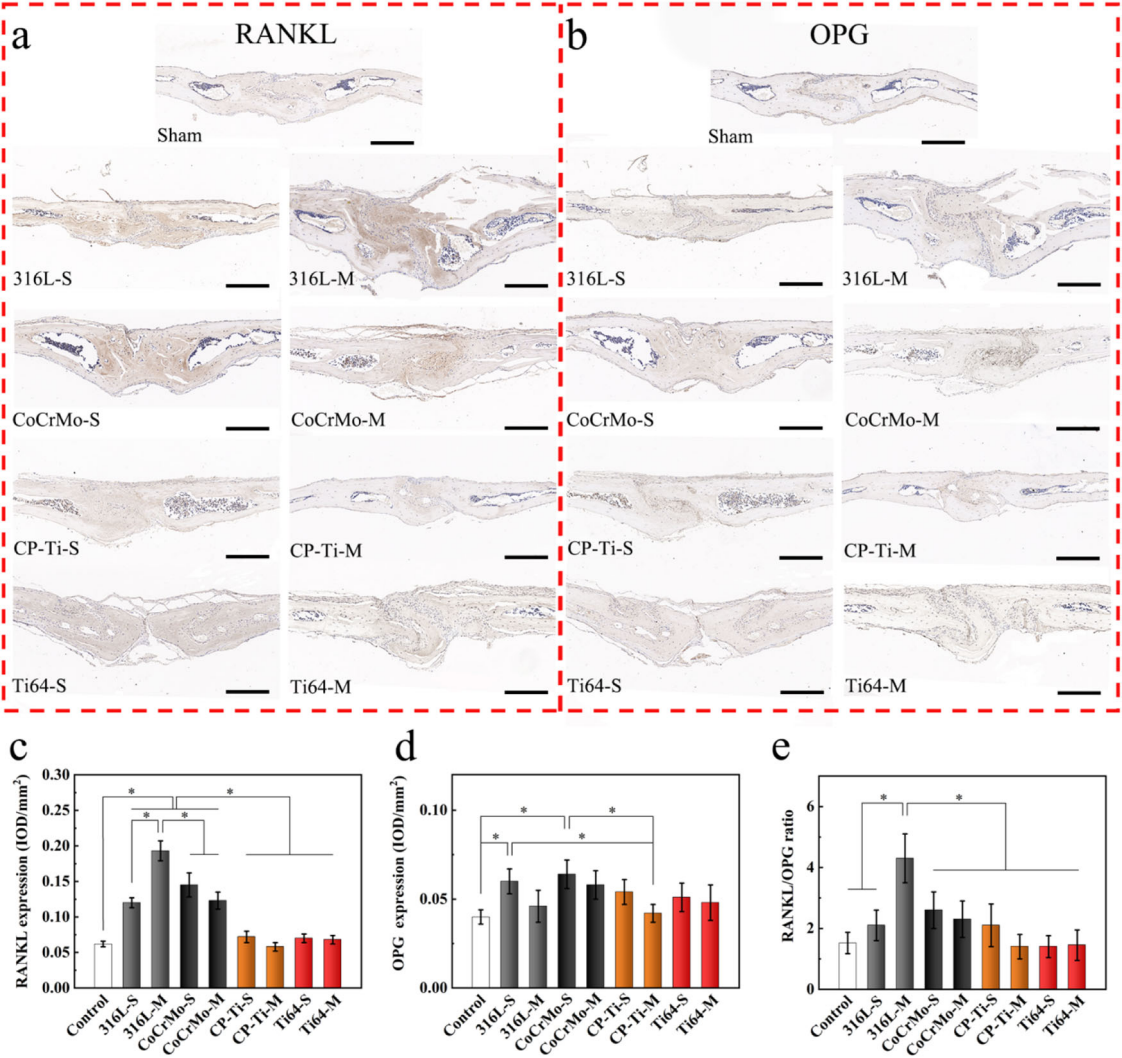
Analysis of RANKL and OPG in skull sections using immunohistochemical staining. Immunohistochemical images of (**a**) RANKL and (**b**) OPG in each group (Scale bar = 200 μm, degree of brown indicates extent of positive staining). The results of semi-quantitative analysis were evaluated by two independent professionals, including (**c**) RANKL expression, (**d**) OPG expression, and (**e**) RANKL/OPG ratio (*p < 0.05)

Immunohistochemical images of the skull tissue are shown in Fig. 8a and b. More RANK-positive cells and dark brown coloration were observed in the RANKL staining sections of the 316L-S, 316L-M, and CoCrMo-S powders. In particular, within the 316L-M group, there were a large number of RANK-positive cells around the severely eroded area (Fig. 9a). The semi-quantitative results of RANKL expression showed that the expression levels of 316L-S, 316L-M, CoCrMo-S, and CoCrMo-M groups were significantly higher than those in the other groups, and the expression level of 316L-M was the highest (Fig. 9c). However, the number of OPG-positive cells, degree of color, and corresponding OPG expression of the sections in the 316L-M group were similar to those in the control group (Fig. 9b and d). Semi-quantitative results of OPG expression showed that the values in the 316L-S and CoCrMo-S groups were significantly higher than those in the control group. Finally, the ratio of RANKL/OPG was found to be significantly higher in the 316L-M group than in any other group, which proves that 316 L powder may induce osteoclast differentiation by regulating the expression of RANKL and OPG, and then trigger inflammation and bone resorption (Fig. 9e).

### Serological analysis of rat femur model

**Fig. 10:**
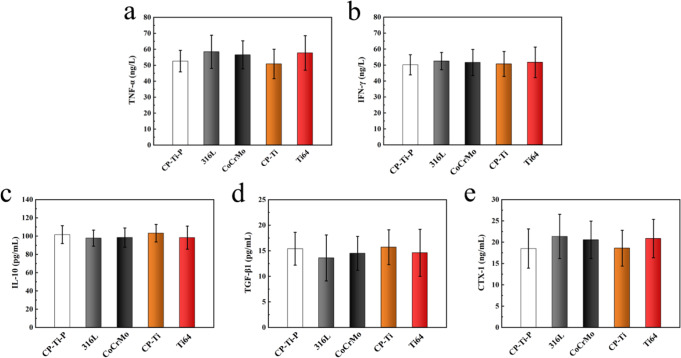
Expression of (**a**) TNF-α, (**b**) IFN-γ, (**c**) IL-10, (**d**) TGF-β1, and (**e**) CTX-1 in serum after implantation for 8 weeks

After implantation for 8 weeks, serum analysis was used to evaluate the immunological response and osteolysis that might have been triggered by the implants with residual powders. It was found that the expression levels of pro-inflammatory and anti-inflammatory factors in all implants were the same, and there was no significant difference, indicating that no immunological response occurred (Fig. 10a–d). CTX-1 is a marker protein of bone resorption in vivo, and changes in its expression have guiding significance for understanding bone resorption [35]. The expression of CTX-1 was the same for all implants and was not significantly different, indicating no evidence of osteolysis (Fig. 10e).

### Osteogenecity of metal implants with residual powders in a rat femoral model

**Fig. 11:**
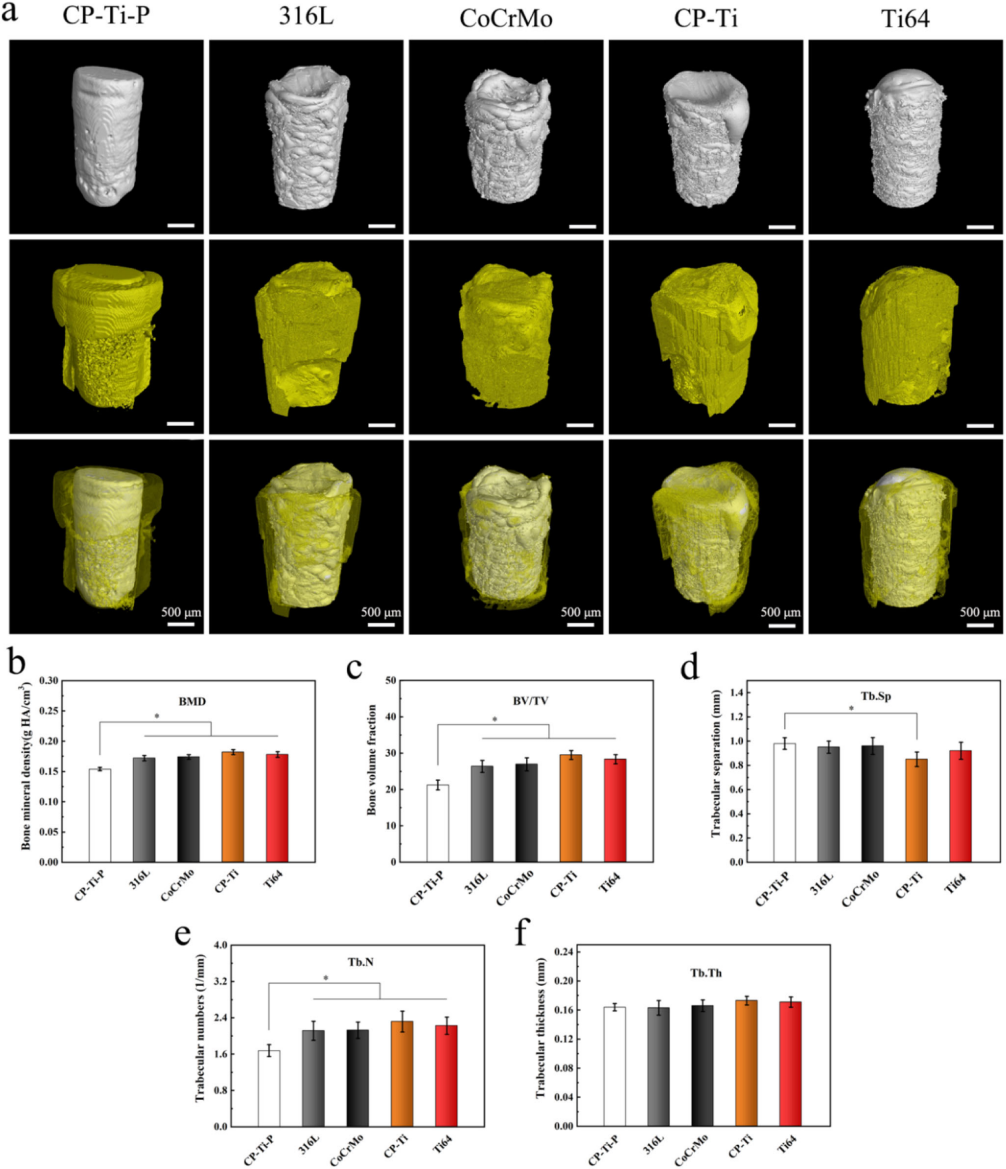
**a** Reconstructed three-dimensional micro-CT images of the implants and new bone formed around the implants after implantation for 8 weeks. Quantitative analyses of osteogenesis parameters, including (**b**) BMD, (**c**) BV/TV, (**d**) Tb.Sp, (**e**) Tb.N, and (**f**) Tb.Th after implantation for 8 weeks (*p < 0.05)

For suitability in clinical practice, histological studies of implants with residual powder should be conducted in vivo. Figure 11a shows the reconstructed micro-CT images of the implants with residual powders and the surrounding bone tissue after implantation for 8 weeks. Due to the difference in bone mineral density between the new bone and the original bone, the new bone is rendered yellow, while the original bone is rendered light yellow during reconstruction. Only a small amount of new bone tissue developed around the CP-Ti-P implant, whereas significantly more new bone tissue extending wider trabecular structures was observed around the 316 L, CoCrMo, CP-Ti, and Ti64 implants, especially around the CP-Ti implant. The quantitative results also showed that the BMD, BV, and Tb.N of all implants with residual powders were significantly higher than those of the polished CP-Ti-P implants (Fig. 11b–d). There was no significant difference in Tb.Th among all groups; however, it is worth noting that the Tb.Sp of the CP-Ti group was significantly lower than that of the other groups (Fig. 11e and f). The above results suggest that implants with residual powders did not cause osteolysis, but showed better osteogenic properties, especially CP-Ti.

**Fig. 12:**
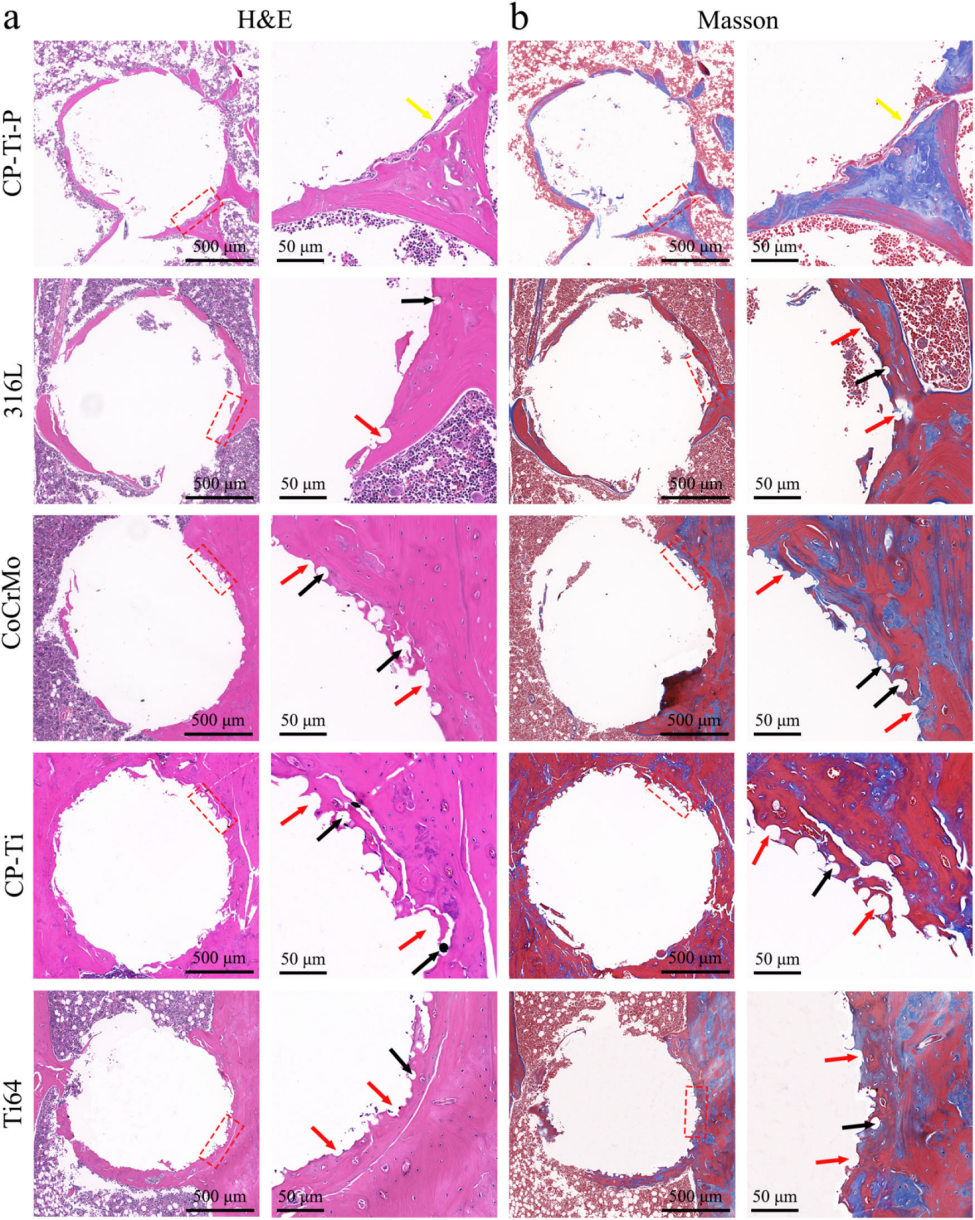
Histological images of the bone defect areas after implantation for 8 weeks, where fibrous capsules (yellow arrows) and semi-circular zigzag interface (red arrows) between the new bone and the implant, and exfoliated powders (black arrows) wrapped by new bone can be observed. Low- and high-magnification images of the bone defect areas are presented from left to right in each set of (**a**) H&E and (**b**) Masson staining images

Histomorphological results showed more new bone formation in the 316 L, CoCrMo, CP-Ti, and Ti64 implants than in the CP-Ti-P implant (Fig. 12). These observations were consistent with the micro-CT results. The fibrous capsule at the interface between the CP-Ti-P implant and the surrounding new bone was observed, and its interface was relatively smooth, showing typical biological inertness. In contrast, the new bone tissue interface of the implants with residual powders had a semi-circular zigzag structure, and no fibrous capsule was observed, showing good staggered osseointegration. It is worth noting that many circular closed vacancies can be observed in the new bone tissue around the implants with residual powder, which is caused by the residual powder being wrapped by the new bone tissue after falling off. Overall, these results further indicate that implants with residual powder did not cause osteolysis but showed better osteogenic properties.

### Distribution of osteoclasts and balance of RNAKL/OPG in a rat femoral model

**Fig. 13:**
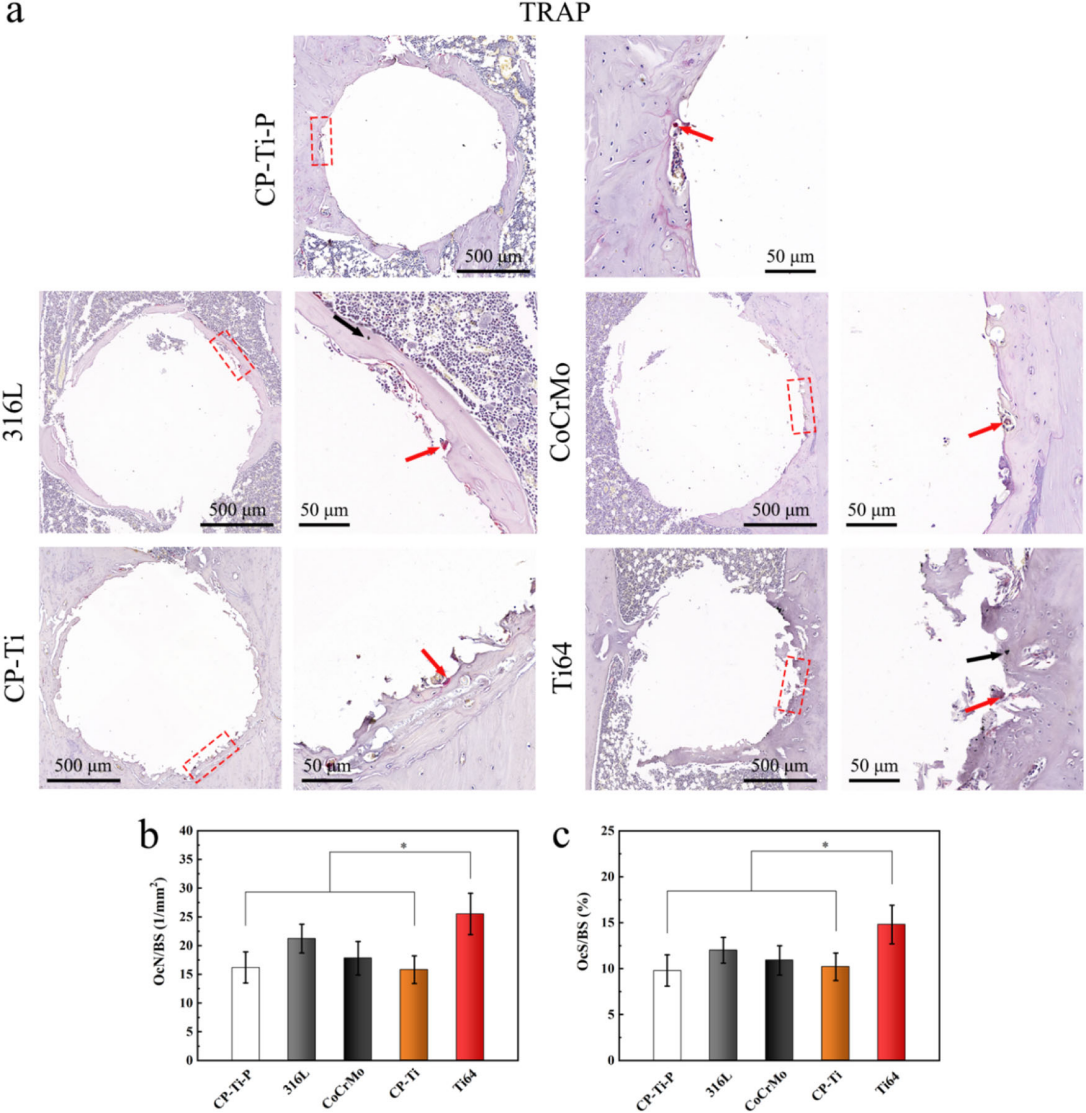
Analysis of osteoclast formation in bone tissue around implants with residual powders. **a** Low- and high-magnification images of the bone defect areas are presented from left to right in each set of representative TRAP staining images, where a small number of osteoclasts (red arrows) and exfoliated powders (black arrows) can be observed. Semi-quantitative results of (**b**) OcN/BS and (**c**) OcS/BS in the ROI of surrounding bone tissue (*p < 0.05)

To confirm that the implant with residual powder did not cause osteolysis in vivo, the distribution of osteoclasts in the surrounding new bone tissue was stained with TRAP, as shown in Fig. 13. A few small-sized powder particles were observed at the interface of the bone tissue in the slices of the 316 L, CoCrMo, and CP-Ti groups, but more in the Ti64 group (Fig. S2, Supplementary Data). Furthermore, more osteoclasts were found in the bone tissue area where small-sized powder particles aggregated. The semi-quantitative results showed that the OcN/BS and OcS/BS values of the Ti64 group were the highest, and there was no significant difference compared with those of the 316 L and CoCrMo groups, but they were significantly higher than those of the CP-Ti-P and CP-Ti groups (Fig. 13b and c).

**Fig. 14:**
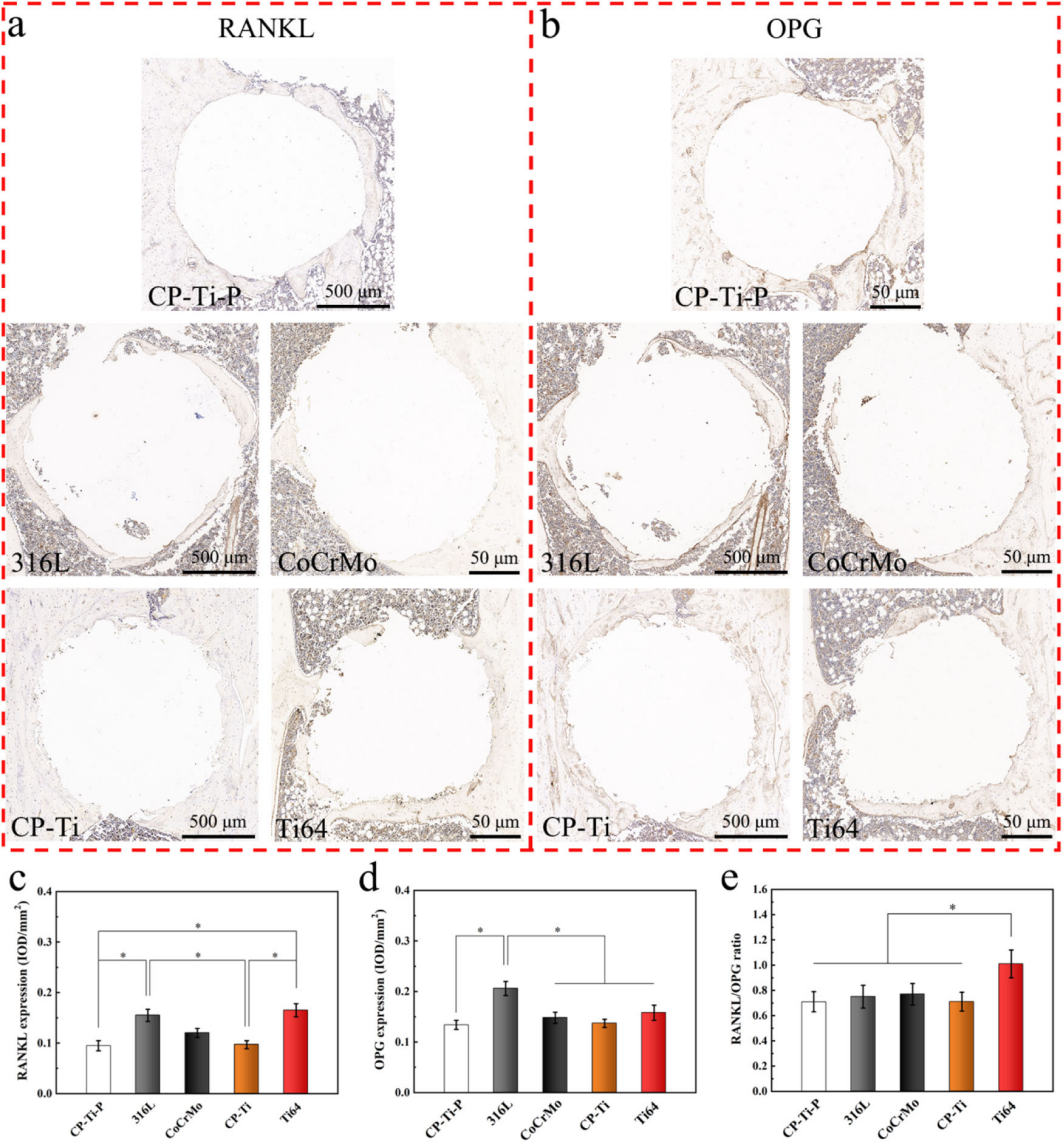
Analysis of RANKL and OPG in the bone defect areas using immunohistochemical staining. Immunohistochemical images of (**a**) RANKL and (**b**) OPG in each group (degree of brown indicates extent of positive staining). Semi-quantitative analysis of the ROI in each group were evaluated by two independent professionals, including (**c**) RANKL expression, (**d**) OPG expression, and (e) RANKL/OPG ratio (*p < 0.05)

As shown in Fig. 14a and b, it was also observed in the immunohistochemical sections that there were a few small-sized powders at the interface of the bone tissue in the 316 L, CoCrMo, and CP-Ti groups, while more powders existed in the Ti64 group. The semi-quantitative results showed that the expression of RANKL in the Ti64 group was the highest and was significantly higher than that in the CP-Ti-P and CP-Ti groups (Fig. 14c). OPG expression was significantly higher in the 316 L group than that in the other groups (Fig. 14d). As the expression levels of both proteins in the 316 L group were relatively high, the RANKL/OPG ratio was almost the same as that of CP-Ti-P (Fig. 14e). However, the proportion of cells in the Ti64 group was slightly higher than that of the other groups, which is consistent with the results of TRAP staining.

## Discussion

### Osteolysis triggered by metal powder

Previously, it has been shown that 3D printing metal powders at a certain concentration (1 × 10^6^ particles/mL) are toxic to immune cells, and the occurrence of immunological responses is related to the morphology, size, and surface chemistry of the powders [24]. To further demonstrate these results, the inflammatory response triggered by prolonged exposure to metal powders in vitro and in vivo must be investigated.

The results of co-culture for 7 days in vitro showed that 316L-M and CoCrMo-S upregulated the secretion of pro-inflammatory factors TNF-α and IFN-γ and downregulated the expression of anti-inflammatory factors, thereby aggravating the inflammatory response. These results were further confirmed by the secretion of inflammatory factors into the mouse skull medium. In addition, 316L-S, 316L-M, and CoCrMo-S powders induced more severe immunological responses and bone resorption than other powders, particularly 316L-M powders. The main reason for this result was closely related to the morphology, size, and surface chemistry of the powder particles. Generally, the smaller the metal particles, the more irregular the particle shape, the higher the biological activity, and the easier it is to induce the release of proinflammatory factors and cause a negative immunological response in the body [36, 37]. In this study, the morphology of the 316L-M powder was irregular, and there were many satellite powders on the surface, which explains why the powder triggered the most severe inflammatory reaction. Although the morphology of the CoCrMo-S powder is highly spherical, there are many nano-satellite powders on their surfaces (Fig. S3, Supplementary Data), indicating that it is easy to promote the occurrence of inflammatory reactions.

Another reason is that the surface chemistry of each powder is different. Fig. S4 shows the X-ray photoelectron spectroscopy (XPS) survey and refined spectra of the CoCrMo-S powders, and it can be considered that the powders were coated by an oxidizing layer primarily composed of Cr_2_O_3_. Our previous studies showed that the surface oxide layer of the 316 L powder was composed of Fe_2_O_3_ and Cr_2_O_3_, whereas the CP-Ti and Ti64 powders were oxide layer TiO_2_. Recent studies have shown that Fe_2_O_3_ and Cr_2_O_3_ are less toxic, and nanoparticles composed of these components are more toxic [38–40]. However, TiO_2_ is often considered an internal environmental substance in the body, and it is difficult to trigger an immunological response [36, 41].

Local inflammatory cell infiltration and osteoclast aggregation play major roles in powder particle-induced osteolysis. After the infiltration of inflammatory cells, more proinflammatory factors are released. Stimulated by these factors, surrounding osteoclasts are recruited, which in turn promotes the differentiation of macrophages and activates them into functional osteoclasts [42]. Osteoclasts are the primary functional cells responsible for bone resorption. Therefore, this study analyzed the distribution of osteoclasts in the area of powder implantation, indicating that osteolysis induced by 316 L and CoCrMo implants is closely related to the activation of osteoclasts.

The RANKL/OPG system is an important pathway for regulating osteoclast activation. Nuclear factor κB receptor activator factor ligand (RANKL) is an activator of NF-κB signaling that can induce osteoclast formation and differentiation, thereby accelerating the inflammatory response. OPG is a competitive inhibitor of RANKL, which inhibits osteoclast activation by preventing RANKL from binding to the receptor RANK on the surface of osteoclast precursor cells [43]. Studies have shown that wear particles can stimulate macrophages, osteoblasts, and fibroblasts in the periprosthetic membrane of loose prostheses to express RANKL and inhibit the release of OPG by osteoblasts, thereby increasing the RANKL/OPG ratio and excessive activation of osteoclasts [44]. In this study, immunohistochemical experiments showed that 316L-M powder upregulated the expression of RNAKL, resulting in a decrease in the RANKL-to-OPG ratio, thereby stimulating the activation of osteoclasts and causing osteolysis. However, the expression of RANKL and OPG and the RANKL/OPG ratio in the Ti powders were similar to those in the control group, and no osteolysis was observed.

### Effects of implant with residual powder in vivo

In actual clinical practice, 3D printed implants are often polished, and some surfaces are post-processed. The purpose is to eliminate the residual and unmelted powders on the surface of the original printed sample, to increase the roughness and biological function of the material, and to strengthen the integration of bone tissue and materials. However, with the increasing understanding of the mechanism of wound healing and the response of host cells to biomedical metal materials, specific “immunoregulatory” metal materials may promote wound healing and osseointegration, which means that residual powders on the surface of 3D printed metal may not be a completely harmful factor [45, 46]. Therefore, the possible immune response and bone regeneration of as-printed implants with residual powders were investigated using an in vivo femur model.

Previous studies have reported that wear particles around the prosthesis cause a local inflammatory response and inhibit body weight gain during animal growth [47, 48]. To be closer to clinical practice, a wear particle-induced loosening model of distal femoral implants was constructed, in which a thick fibrous capsule was formed between the loosened prosthesis and cancellous or cortical bone [31]. Wear particles usually exist in the fibrous capsule, which is caused by the reaction of lymphocytes [49, 50]. Persistently, it causes an increase in osteoclasts around the prosthesis, accelerates the dissolution of surrounding bone, and ultimately leads to the failure of prosthesis implantation [51]. In this study, the residual powder on the implant surface effectively promoted the integration of bone tissue, which is different from prosthesis loosening caused by wear particles in vivo.

Osteolysis is the direct result of bone tissue inflammation. 3D micro-CT reconstruction and analysis of the bone tissue around the implants with residual powders showed that this phenomenon did not occur but promoted bone integration, especially the CP-Ti implant. Furthermore, inflammatory cytokines and CTX-1 are markers of bone resorption caused by increased osteoclast activity, which can reflect the level of bone turnover in vivo [35, 52]. It was found that implants with residual powder did not cause high expression of pro-inflammatory cytokines and CTX-1 in the serum. It is worth noting that there were many small-sized powder particles at the bone tissue interface around the Ti64 implant, and more osteoclasts were observed near the powder particles. Small-sized powder particles are likely to exist in the gap between the residual powders or partially melted powders of the Ti64 implant, and are not cleaned during ultrasonic cleaning. After implantation, small-sized powder particles in the gap caused slight inflammation and osteoclast aggregation after exposure in vivo. RANKL and OPG are considered the most important factors in osteoclast formation, and their balance plays an important role in osteoclast differentiation and function [53]. Analysis of the protein expression of the two factors in the bone tissue around the implant showed that the Ti64 group upregulated the expression of RANKL and increased the RANKL/OPG ratio, which also indicated that the small-sized powder particles exposed on the implant surface promoted slight inflammation. In the long term, Ti64 implants do not have bone resorption at 8 weeks but may have some negative effects as inflammation continues.

The above results indicate that implants with residual powder do not lead to serious inflammation but promote bone regeneration and integration. This is mainly due to its residual powder giving the implant a micron-scale roughness, which is more conducive to the growth of bone tissue. If large-sized powder particles (≥10 µm) fall off, they will not lead to an inflammatory reaction. In contrast, the dissociation of small-sized powder particles is the main cause of inflammatory reactions. By comparing the experimental results of the skull model, the results of the femoral model are optimistic and instructive for bone tissue regeneration and repair. The main reasons for the difference between the results of the two models include that:

1. The residual powders on the surface of the 3D printed implant are rougher and more spherical than the raw powders of 3D printing because the heat-affected powders collide with each other and aggregate during the melting and solidification of the printing [54]. The biosafety of powder particles with larger particle size and better sphericity is excellent in vivo, and the powder particles bring micron-scale roughness to the implant, accelerating the growth and adhesion of osteoblasts.
2. There are more satellite powders on the surface of the raw powders, which promotes the occurrence of inflammatory reactions. However, the amount of it glued to the surface of the printed implant is relatively less, and it is further reduced during ultrasonic cleaning.
3. The oxygen content on the surface of the residual powder is higher than that of the original powder because of its slower cooling rate in the atmosphere of the building chamber [55]. The increase of oxygen content is helpful to improve the biosafety of materials.

Therefore, this means that the application of 3D printed solid implants in clinical practice does not need to deliberately remove the residual powders on the surface but only needs to clean up the small-sized particles on the surface and some loose powder that is easy to fall off. However, for 3D printed porous implants, especially those with small pore size, there may be more satellite powder inside and it is difficult to remove, which may lead to counterproductive results. In addition, by preserving the roughness of the as-printed implants, appropriate surface modifications may have great potential in clinical applications.

## Conclusions

To better understand the potential effects of various metal powders on the immune and bone systems, this study systematically explored the immunological responses and osteogenesis triggered by four typical metal powders (316 L, CoCrMo, CP-Ti, and Ti64) using skull and femur models in vivo. 316L-S, 316L-M, and CoCrMo-S powders, especially 316L-M powders, upregulated the expression of inflammatory factors, disrupted the balance of RANKL/OPG expression, promoted osteoclast formation, and eventually led to severe skull osteolysis. However, for implants with residual powders, especially the CP-Ti implant, no bone absorption was found, but bone integration was promoted. As-printed implants with micron roughness are considered to have a greater potential for clinical applications.

## Supplementary information

Supplementary Information
