# Supplementary material for: Impacts of residual 3D printing metal powders on immunological response and bone regeneration: an in vivo study
Source: J Mater Sci Mater Med. 2023 May 25;34(6):29. doi: 10.1007/s10856-023-06727-1 (PMC10212846; doi:10.1007/s10856-023-06727-1)
Supplement: Supplementary file 1 — Supplementary Information [file 10856_2023_6727_MOESM1_ESM.docx]

**Supporting Information for**

**Impacts of residual 3D printing metal powders on immunological response and bone regeneration: An *in vivo* study**

Jincheng Tang^a^, Zhuo Sang^b,*^, Xiaolei Zhang^b^, Changhui Song^c^, Wei Tang^d^, Xiaoping Luo^e^, Ming Yan^a,*^

^a^Department of Materials Science and Engineering, Southern University of Science and Technology, Shenzhen, 518055, China

^b^The Eighth Affiliated Hospital, Sun Yat- sen University, Shenzhen, 518033, China

^c^Department of Mechanical and Automotive Engineering, South China University of Technology, Guangzhou 510641, China

^d^Shenzhen Institutes of Advanced Technology, Chinese Academy of Sciences, Shenzhen, 518055, China

^e^Nanjing Stomatological Hospital Medical School of Nanjing University, Nanjing 210008, China

*Corresponding authors, E-mail addresses: [yanm@sustech.edu.cn](mailto:yanm@sustech.edu.cn); sangzhuohao@163.com


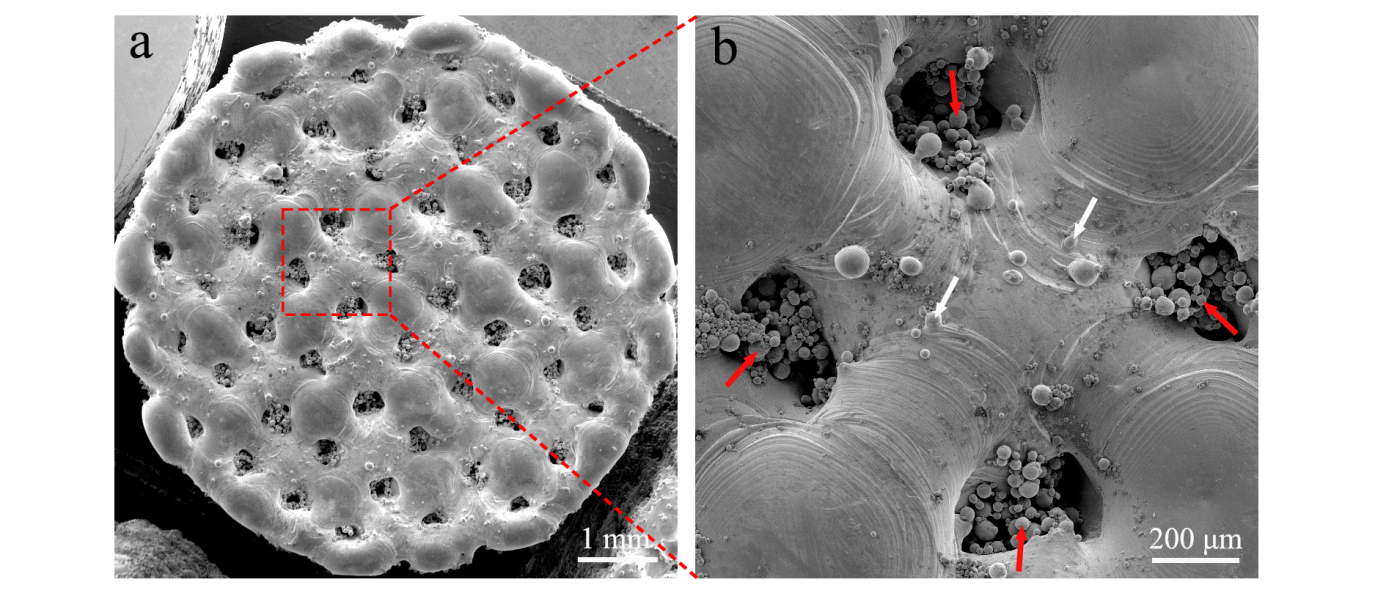


Fig. S1 (a) SEM micrograph of SLM-printed Ti-15Mo lattice. (b) Enlarged image of the red square area form (a), where partially-melted powders (white arrows) and loose powders (red arrows) on the surface of the implants can be observed.

Table S1 SLM processing parameters of all implants.

| Implants | Power (W) | Scanning  speed  (mm/s) | Layer  thickness  (μm) | Hatching  space  (μm) | Energy  density  (J/mm^3^) |
| --- | --- | --- | --- | --- | --- |
| 316L | 200 | 800 | 30 | 120 | 69.44 |
| CoCrMo | 360 | 500 | 50 | 175 | 82.29 |
| CP-Ti | 176 | 650 | 30 | 100 | 90.26 |
| Ti64 | 176 | 650 | 30 | 100 | 90.26 |


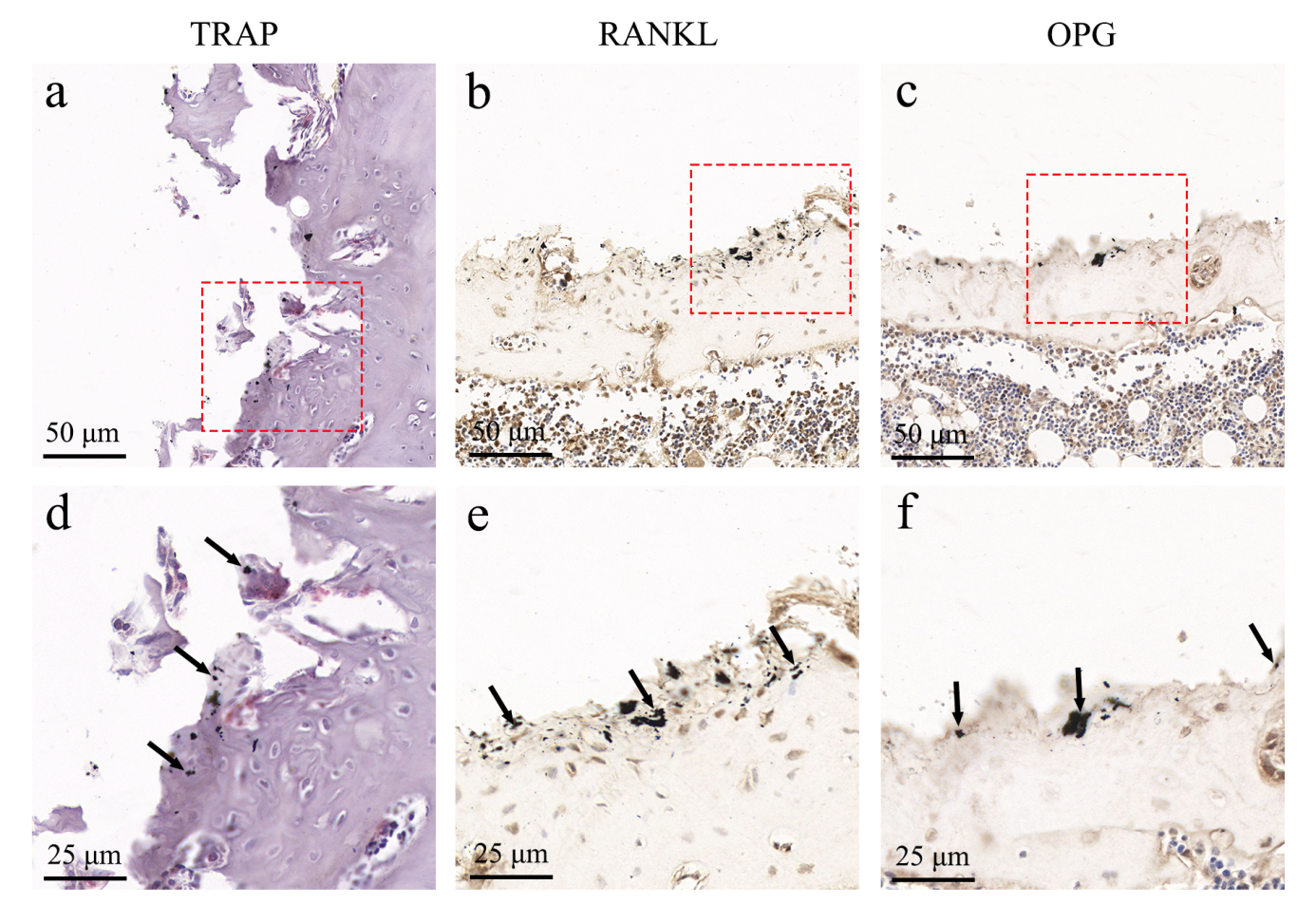


Fig. S2 (a-c) High-magnification staining images of bone tissue around the Ti64 implant. (d-f) Enlarged images of the red square area from the staining images, where more small-sized powder particles (black arrows) can be observed.

**
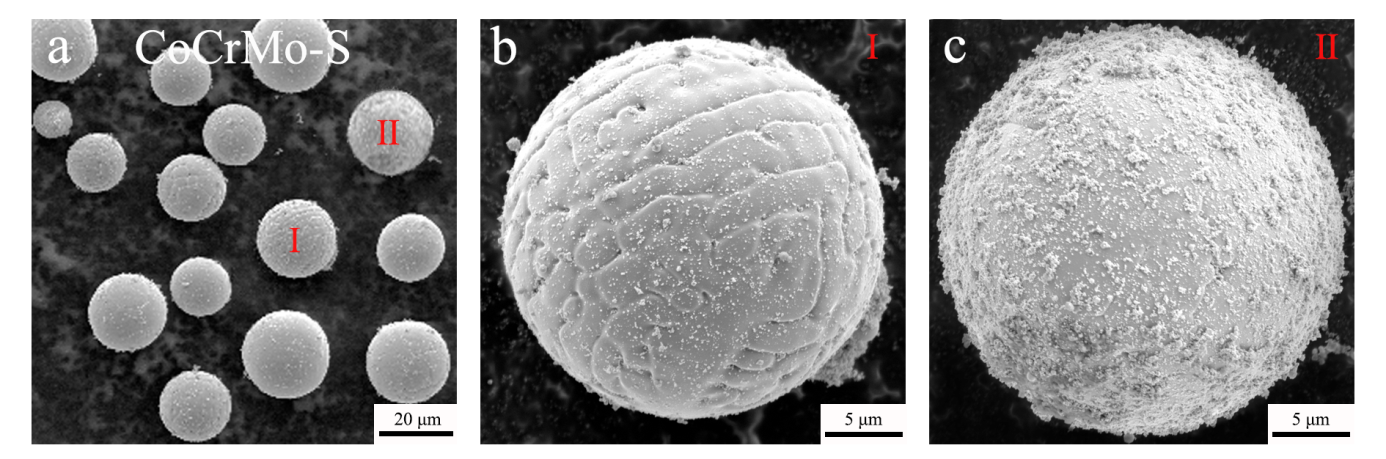
**

Fig. S3 (a) SEM image of the CoCrMo-S powder. (b,c) Enlarged images of single powder particles corresponding to areas Ⅰ and Ⅱ in (a), repectively.


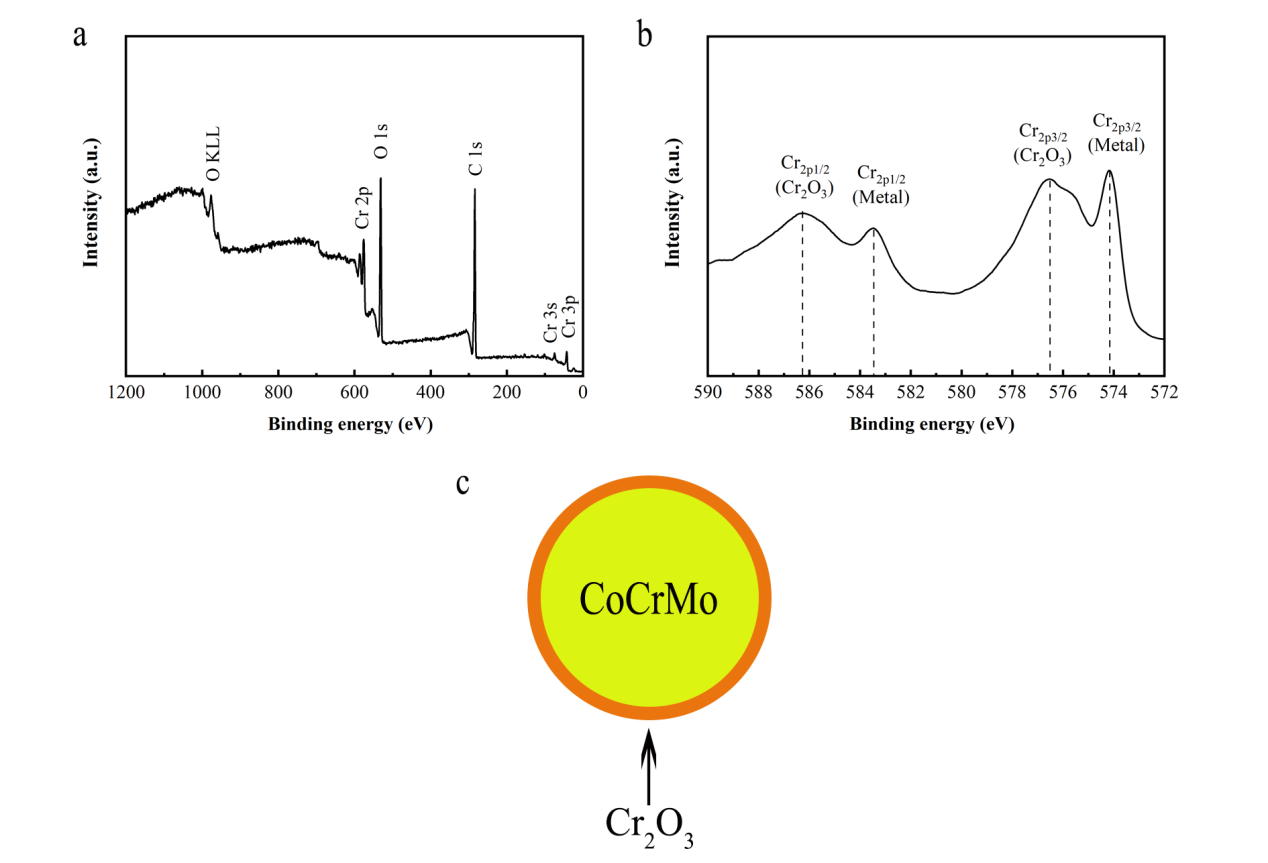


Fig. S4 (a) XPS survey spectrum and (b) detailed XPS spectra of Cr on the surface of the CoCrMo powders. (C) Schematic of the surface structure of the 316L-S powder.
